# S7-2 From HEPA to sustainable HEPA?

**DOI:** 10.1093/eurpub/ckad133.034

**Published:** 2023-09-11

**Authors:** Karim Abu-Omar, Peter Gelius, Sven Messing, Antonina Tcymbal, Viktoria Kovacs

**Affiliations:** Friedrich-Alexander Universität Erlangen-Nürnberg; Friedrich-Alexander Universität Erlangen-Nürnberg; Friedrich-Alexander Universität Erlangen-Nürnberg; Friedrich-Alexander Universität Erlangen-Nürnberg; WHO European Office for Prevention and Control of Noncommunicable Diseases

## Abstract

**Purpose:**

Current climate change poses a challenge to physical activity (PA) promotion. Not only is PA more difficult in heat and people need to adapt, but also many physical activities come with a comparable high carbon footprint. While the field of PA promotion has so far utilized the health-enhancing physical activity (HEPA) concept to define and promote PA, it might be asked if this concept needs to be amended in order to account for the environmental impact of PA. The aim of the presentation is to critically analyze existing concepts of PA promotion and also healthy diet and suggest potential steps towards a concept of sustainable HEPA.

**Methods:**

To deal with the difficult question on how to consider climate change in non-communicable disease prevention, the WHO Europe hosted an expert meeting in the fall of last year on which the concepts of sustainable diet and sustainable PA were discussed. Additionally, some articles and reviews related to interactions between PA and planetary health were screened, and international PA documents reviewed.

**Results:**

The HEPA model is based on a bio-medical model of health and represents the best available evidence from epidemiological studies on the health effects of PA. This model is very well suited to promote PA among individuals, but tends to ignore the environmental impacts of people engaging in PA. A concept of sustainable HEPA that draws on the concept of sustainable diet would allow to account for the environmental impact of PA besides the health effects. However, the development of such a concept would also in all likelihood results in more complex messages about what type and how much PA people should do for themselves and for the public.

**Conclusions:**

There might be a need to develop a new concept for PA promotion. In order to do so, however, a number of ethical and also practical questions would need to be answered. Potentially, collaborating closely with the field of nutrition and drawing on the experiences they made in developing the concept of sustainable diet can be helpful to overcome some of these challenges.

**Support/Funding Source:**

no outside funding.

